# Outcomes After Early Pregnancy Loss Management With Mifepristone Plus Misoprostol vs Misoprostol Alone

**DOI:** 10.1001/jamanetworkopen.2024.35906

**Published:** 2024-10-08

**Authors:** Lyndsey S. Benson, Navya Gunaje, Sarah K. Holt, John L. Gore, Vanessa K. Dalton

**Affiliations:** 1 Department of Obstetrics and Gynecology, University of Washington School of Medicine, Seattle; 2 Department of Urology, University of Washington School of Medicine, Seattle; 3 Department of Obstetrics and Gynecology, University of Michigan, Ann Arbor

## Abstract

**Importance:**

Medication management of early pregnancy loss (EPL), or miscarriage, typically involves the administration of misoprostol with or without pretreatment with mifepristone. Combination treatment with mifepristone plus misoprostol is more effective than misoprostol alone but is underutilized in the US.

**Objective:**

To describe differences in clinical outcomes after EPL management with mifepristone plus misoprostol vs misoprostol alone using commercial claims data.

**Design, Setting, and Participants:**

This retrospective cohort study used national insurance claims data from the IBM MarketScan Research Database. Participants included pregnant people (aged 15-49 years) with private insurance who presented with an initial EPL diagnosis between October 1, 2015, and December 31, 2022.

**Exposures:**

The primary exposure was the medication used to manage EPL (ie, mifepristone plus misoprostol or misoprostol alone). Other exposures of interest included demographic characteristics and location of service.

**Main Outcomes and Measures:**

The primary outcome was subsequent procedural management (eg, uterine aspiration) after EPL diagnosis and medication management. Other outcomes of interest included return visits, hospitalizations, and complications occurring in the subsequent 6 weeks. Descriptive statistics and bivariate analyses were used, and a multivariable logistic regression model was created to examine factors associated with subsequent procedural management.

**Results:**

This study included 31 977 patients (mean [SD] age, 32.7 [5.6] years) with claims for EPL who received medication management. Of these patients, 3.0% received mifepristone plus misoprostol and 97.0% received misoprostol alone. Patients who received misoprostol with pretreatment with mifepristone were less likely to have subsequent uterine aspiration (10.5% vs 14.0%; *P* = .002), and they were also less likely to have subsequent emergency department (ED) visits for EPL (3.5% vs 7.9%; *P* < .001). In multivariable analysis, use of mifepristone plus misoprostol was associated with decreased odds of subsequent procedural management (adjusted odds ratio, 0.71 [95% CI, 0.57-0.87]).

**Conclusions and Relevance:**

The findings of this study suggest that mifepristone is underutilized for the medication management of EPL, but its use is associated with a lower need for subsequent uterine aspiration and a decrease in the number of subsequent visits to an ED. Increasing access to mifepristone for EPL management may decrease health care utilization and expenditures.

## Key Points

**Question:**

How do outcomes differ when patients experiencing early pregnancy loss (EPL) receive mifepristone plus misoprostol vs misoprostol alone?

**Findings:**

In this cohort study of 31 977 patients, management of EPL with misoprostol alone was associated with increased incidence of subsequent procedural management and increased emergency department visits compared with mifepristone plus misoprostol combination treatment.

**Meaning:**

These findings suggest that mifepristone remains underused for EPL management and that increased access to mifepristone for EPL management may decrease health care utilization and expenditures.

## Teaser

This cohort study uses commercial claims data to examine differences in outcomes after early pregnancy loss management with mifepristone plus misoprostol compared with misoprostol alone.

## Introduction

Early pregnancy loss (EPL), or miscarriage, is the most common complication of early pregnancy and affects more than 1 million people in the US each year.^1,2^ Early pregnancy loss is defined as a nonviable, intrauterine pregnancy at less than 13 weeks of gestation.^1^ Treatment options for patients experiencing EPL include expectant management, medication management, and procedural management (eg, uterine aspiration).^1^ For patients who are hemodynamically stable, all 3 options are typically reasonable, and management should be guided using shared decision-making. Recent evidence suggests that approximately 10% of US patients with an EPL diagnosis receive medication management, but the proportion of patients who were offered or received their preferred management option remains unknown.^3^

Medication management of EPL typically includes the administration of misoprostol with or without pretreatment with mifepristone.^1^ Combination treatment with mifepristone plus misoprostol has been shown in clinical trials to be more effective than misoprostol alone for EPL management.^4,5^ In 1 randomized clinical trial, the addition of mifepristone increased efficacy of medication management from 76% to 91%.^4^ Patients for whom medication management is not successful will commonly undergo subsequent procedural management (eg, uterine aspiration).^1^ Despite updated guidance from the American College of Obstetricians and Gynecologists (ACOG) in 2018, mifepristone remains underutilized for patients with EPL.^1,6^ Mifepristone is highly regulated and there are substantial barriers to its use, in large part due to its use for medication abortion.^7,8^ We sought to describe differences in clinical outcomes after EPL management with mifepristone plus misoprostol vs misoprostol alone using commercial claims data.

## Methods

### Study Population and Data

We performed a retrospective cohort study of pregnant individuals who experienced EPL in the US between October 1, 2015, and December 31, 2022, using the IBM MarketScan Research Database. This database comprises deidentified claims data from commercially insured US patients obtained from a large convenience sample of employers and health plans that agree to participate. Available data include dates and locations of service, demographic characteristics, diagnosis codes, procedure codes, and medication prescriptions. This study was deemed exempt from review and the need for informed consent by the University of Washington institutional review board owing to the use of deidentified data. The study followed the Strengthening the Reporting of Observational Studies in Epidemiology (STROBE) reporting guideline.

We included individuals aged 15 to 49 years with a diagnosis of EPL, which was defined using *International Classification of Diseases, Tenth Revision, Clinical Modification* (*ICD-10-CM*) diagnosis codes O02.1 (missed abortion) and O03.x (spontaneous abortion) (eTable in Supplement 1). Eligible patients were required to have available claims data for 90 days before and 60 days after the index EPL diagnosis and to have prescription data available. For patients with multiple pregnancy losses, only the first EPL episode was included in this cohort. We limited our sample to patients with evidence of medication management, defined as having a claim for misoprostol with or without mifepristone in the 7 days after an index EPL diagnosis, without evidence of a uterine aspiration procedure during that same time period.

We excluded patients who had a concurrent diagnosis of ectopic or molar pregnancy, induced abortion, or stillbirth or who had evidence of recent EPL management (either with medication or uterine aspiration). To exclude patients who likely had a pregnancy diagnosis other than EPL, we used the validated algorithm developed by Matcho et al^9^ to infer pregnancy episodes and outcomes. Patients with evidence of recent EPL management were excluded if they had a prescription for mifepristone or misoprostol or underwent uterine aspiration within 56 days before their index EPL diagnosis; this was the minimum length of time to be considered a separate pregnancy episode per the algorithm developed by Matcho et al.^9^

### Measures

The primary exposure of interest was receiving mifepristone plus misoprostol vs misoprostol alone. Additional covariates of interest included age (in years), encounter year, US geographic region (Northeast, North Central, South, or West), metropolitan statistical area status (rural vs urban location), location of service (outpatient clinic, emergency department [ED], or other), and insurance policy status (primary policy holder or spouse vs dependent). We included encounter year as a binary variable (2015-2018 vs 2019-2022) in our multivariable model, given the new data and updated ACOG guidance published in 2018 that supported the use of mifepristone in medication management of EPL.^1,4^

This was an exploratory analysis with outcomes of interest including need for subsequent procedural management (ie, uterine aspiration after the initial 7 days following EPL diagnosis), return visits to the ED or outpatient clinic setting, hospitalizations, and complications occurring in the subsequent 6 weeks after initial EPL diagnosis. Complications included hemorrhage requiring blood transfusion, uterine artery embolization, hysterectomy or other surgical management with laparotomy or laparoscopy, cervical laceration, uterine perforation or other genitourinary tract injury, and intrauterine infection. Because of the low rates of complications overall, we created a composite complication variable that included hospitalization related to EPL, blood transfusion, and hysterectomy or other surgical management apart from uterine aspiration. All outcomes and complications were determined through *ICD-10-CM* diagnosis codes, *ICD-10-CM* procedure codes, and *Current Procedural Terminology* codes (eTable in Supplement 1).

### Statistical Analysis

Demographic characteristics with descriptive statistics are reported. We conducted bivariate analyses to explore associations between demographic characteristics and medication management type (mifepristone plus misoprostol vs misoprostol alone), using χ^2^ tests for categorical variables and *t* tests for continuous variables. We also conducted bivariate analyses to investigate associations between type of medication management and outcomes and complications. We then created a multivariable logistic regression model to examine factors associated with subsequent procedural management after initial medication management of EPL. The primary indicator of interest was medication management type (mifepristone plus misoprostol vs misoprostol alone), and we adjusted for available demographic characteristics, encounter year, and location of service. We used the Hosmer-Lemeshow goodness-of-fit test to assess the fit of our logistic regression model. Given concerns for variable access to and insurance coverage for mifepristone, we planned a priori to complete a sensitivity analysis including only states with Medicaid insurance coverage of abortion, as we expected mifepristone coverage to be more complete in these states. Two-tailed *P* < .05 was considered statistically significant. All analyses were completed with SAS, version 9.4 M8 (SAS Institute Inc).

## Results

We identified 31 977 patients (mean [SD] age, 32.7 [5.6] years) who received medication management following a diagnosis of EPL (Figure). The proportion of individuals who received mifepristone plus misoprostol increased from 0.7% (7 of 1053) in 2015 to 8.6% (357 of 4149) in 2022 (*P* < .001; Table 1). Similar to previous findings,^6^ mifepristone use was more common among older patients (mean [SD] age, 33.8 [4.9] vs 32.6 [5.6] years), in urban settings (3.2% [822 of 26 036] vs 2.5% [144 of 5650]), and in the Northeast (8.9% [428 of 4810]) and West (5.5% [347 of 6346]) vs the North Central (1.5% [82 of 5304]) and South (0.7% [108 of 15 205]) regions (all *P* < .001). The majority of patients (72.3%) had a diagnosis of missed abortion, 26.9% had a diagnosis of spontaneous abortion, and 0.8% had both diagnosis codes. Among patients with a missed abortion diagnosis, 3.3% received mifepristone; among those with a spontaneous abortion diagnosis, 2.5% received mifepristone.

**Figure.**
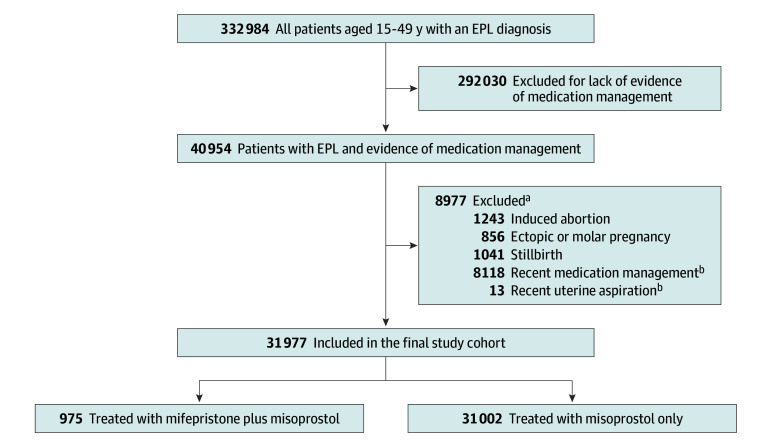
Patient Selection Flowchart ^a^Some patients were excluded for more than 1 reason. ^b^Patients who had evidence of recent early pregnancy loss (EPL) management were excluded if they had a prescription for mifepristone or misoprostol or underwent uterine aspiration within 56 days before their index EPL diagnosis; this was the minimum length of time to be considered a separate pregnancy episode. This effort was made to exclude patients for whom this index EPL diagnosis was not likely a new diagnosis or pregnancy.

**Table 1.** Patient Characteristics^a^

Characteristic	All patients (N = 31 977)	EPL management		*P* value
		Mifepristone plus misoprostol (n = 975)	Misoprostol only (n = 31 002)	
Age, mean (SD), y	32.7 (5.6)	33.8 (4.9)	32.6 (5.6)	<.001
Encounter year				
2015^b^	1053 (3.3)	7 (0.7)	1046 (3.4)	<.001
2016	4059 (12.7)	23 (2.4)	4036 (13.0)	
2017	4161 (13.0)	33 (3.4)	4128 (13.3)	
2018	4822 (15.1)	50 (5.1)	4772 (15.4)	
2019	4845 (15.2)	73 (7.5)	4772 (15.4)	
2020	4371 (13.7)	151 (15.5)	4220 (13.6)	
2021	4517 (14.1)	281 (28.8)	4236 (13.7)	
2022	4149 (13.0)	357 (36.6)	3792 (12.2)	
US geographic region				
Northeast	4810 (15.0)	428 (43.9)	4328 (14.0)	<.001
North Central	5304 (16.6)	82 (8.4)	5222 (16.8)	
South	15 205 (47.6)	108 (11.1)	15 097 (48.7)	
West	6346 (19.9)	347 (35.6)	5999 (19.4)	
Metropolitan statistical area				
Rural	5650 (17.7)	144 (14.8)	5506 (17.8)	.02
Urban	26036 (81.4)	822 (84.3)	25 214 (81.3)	
Location of service				
Outpatient clinic	26 635 (83.3)	902 (92.5)	25 733 (83.0)	<.001
Emergency department	3412 (10.7)	31 (3.2)	3381 (10.9)	
Other	1930 (6.0)	42 (4.3)	1888 (6.1)	
Insurance coverage				
Primary or spouse	29 736 (93.0)	939 (96.3)	28 797 (92.9)	<.001
Dependent	1950 (6.1)	27 (2.8)	1923 (6.2)	

Abbreviation: EPL, early pregnancy loss.

^a^ Unless indicated otherwise, values are presented as No. (%) of patients. Some proportions do not sum to 100% due to missing data.

^b^ The study period was from October 1, 2015, to December 31, 2022; 2015 proportions are smaller because there are data from only 3 months in 2015.

The majority of patients (83.3%) received their initial diagnosis of EPL in an outpatient clinic, whereas others initially presented to an ED (10.7%) or other setting (6.0%). We found that mifepristone use was higher when the index EPL diagnosis occurred in the outpatient vs ED setting (3.4% vs 0.9%; *P* < .001). Misoprostol-only management was associated with increased incidence of subsequent procedural management (14.0% vs 10.5%; *P* = .002) and increased ED visits (7.9% vs 3.5%; *P* < .001; Table 2). Among the subgroup of patients who had an initial EPL diagnosis in an outpatient clinic rather than the ED, those who received misoprostol only were more than twice as likely to have subsequent EPL-related care in the ED compared with those who received mifepristone plus misoprostol (7.1% vs 3.1%; *P* < .001). Complications were low overall, with 0.9% composite complications in the misoprostol-only group and 0.4% composite complications in the mifepristone plus misoprostol group (*P* = .10).

**Table 2.** Outcomes and Complications After EPL Management With and Without Mifepristone^a^

Outcome or complication	All patients (N = 31 977)	EPL management		*P* value
		Mifepristone plus misoprostol (n = 975)	Misoprostol only (n = 31 002)	
Outcome				
Subsequent procedural management^b^	4455 (13.9)	102 (10.5)	4353 (14.0)	.002
Subsequent medication management^c^	175 (0.6)	5 (0.5)	170 (0.6)	.88
Inpatient hospitalization, EPL related^d^	163 (0.5)	2 (0.2)	161 (0.5)	.18
Subsequent ED visits, EPL related^d^	2472 (7.7)	34 (3.5)	2438 (7.9)	<.001
Subsequent outpatient clinic visits, EPL related^d^	23628 (73.9)	720 (73.8)	22908 (73.9)	.97
Complication^d^				
Hemorrhage requiring blood transfusion	107 (0.3)	0	107 (0.4)	NA
Uterine artery embolization	3 (0.01)	0	3 (0.01)	NA
Hysterectomy	5 (0.02)	0	5 (0.02)	NA
Other surgical management (laparotomy or laparoscopy)	10 (0.03)	0	10 (0.03)	NA
Cervical injury or laceration repair	2 (0.01)	0	2 (0.01)	NA
Intrauterine infection	157 (0.5)	4 (0.4)	153 (0.5)	.71
Uterine perforation or other genitourinary tract injury	9 (0.03)	0	9 (0.03)	NA
Composite complication outcome	287 (0.9)	4 (0.4)	283 (0.9)	.10

Abbreviations: ED, emergency department; EPL, early pregnancy loss; NA, not applicable.

^a^ Unless indicated otherwise, values are presented as No. (%) of patients.

^b^ Defined as evidence of a uterine aspiration at days 8 to 42 after index EPL diagnosis, because treatment within days 1 to 7 was deemed primary EPL management.

^c^ Defined as provision of misoprostol with or without mifepristone at days 8 to 42 after index EPL diagnosis, because treatment within days 1 to 7 was deemed primary EPL management.

^d^ Included if occurred in the 42 days after the index EPL diagnosis.

In our multivariable analysis, mifepristone use was associated with decreased odds of subsequent procedural management (adjusted odds ratio [AOR], 0.71 [95% CI, 0.57-0.87]; Table 3). Additional factors associated with decreased odds of procedural management included having an initial visit in the ED (AOR, 0.62 [95% CI, 0.55-0.69]) and being a dependent rather than primary policy holder (AOR, 0.81 [95% CI, 0.69-0.95]). Our Hosmer-Lemeshow goodness-of-fit statistic for this model had a *P* value of .12. In our sensitivity analysis looking only at states with Medicaid coverage for induced abortion, our findings were unchanged. This sensitivity analysis included data from 9321 individuals (29.1% of our total cohort). Within this subgroup, the association between mifepristone use and odds of subsequent procedural management was similar to our overall cohort (AOR, 0.67 [95% CI, 0.51-0.87]).

**Table 3.** Factors Associated With Need for Procedural Management After Initial Medication Management for Early Pregnancy Loss

Factor	AOR (95% CI)
Mifepristone plus misoprostol (vs misoprostol alone)	0.71 (0.57-0.87)
Age, y	0.99 (0.99-1.00)
Encounter year (2019-2022 vs 2015-2018)	1.03 (0.99-1.03)
US geographic region	
Northeast	1.08 (0.97-1.21)
North Central	1.23 (1.10-1.36)
South	1.11 (1.02-1.21)
West	1 [Reference]
Metropolitan statistical area	
Rural	1 [Reference]
Urban	0.99 (0.91-1.07)
Location of service	
Outpatient clinic	1 [Reference]
Emergency department	0.62 (0.55-0.69)
Other	0.85 (0.74-0.97)
Insurance coverage	
Primary or spouse	1 [Reference]
Dependent	0.81 (0.69-0.95)

Abbreviation: AOR, adjusted odds ratio.

## Discussion

In this retrospective analysis of privately insured patients, we found differences in outcomes of patients receiving mifepristone plus misoprostol vs misoprostol alone for EPL management. Patients who received misoprostol alone without pretreatment with mifepristone were more likely to receive subsequent procedural management and to have subsequent visits to the ED. These differences are consistent with findings from clinical trials^4,5^ and suggest a greater burden on patients and the health care system (in terms of additional procedures and follow-up care) when mifepristone is not utilized.

Although mifepristone use increased significantly throughout the study period, it remained substantially underutilized, with only 8.6% of patients receiving mifepristone for medication management of EPL in 2022 despite high-quality evidence and society guidelines supporting its use. During this same time period, mifepristone use in the setting of first-trimester abortion increased significantly, with medication abortion (typically accomplished with mifepristone and misoprostol) now accounting for two-thirds of first-trimester abortions in the US.^10^ The continued underutilization of mifepristone in the setting of EPL is multifactorial, with prior studies identifying multiple barriers to its use, including logistical barriers and uncertainty related to US Food and Drug Administration Risk Evaluation and Mitigation Strategy requirements, resistance from institutional leadership, and lack of education or prior experience with mifepristone.^11,12,13^

We also found that rates of mifepristone use were statistically significantly lower when the index diagnosis of EPL occurred in an ED. Although EPL management, particularly procedural management, may not always be feasible in an ED setting, there is an opportunity to improve care for patients opting for medication management. These improvements could include better linkage to subsequent care after an EPL diagnosis in the ED (our definition of medication management included patients receiving misoprostol with or without mifepristone in the 7 days after index EPL diagnosis) or ideally access to and provision of these medications in the ED setting. Despite lower use of mifepristone for patients seen in the ED, they were less likely to need subsequent procedural management compared with patients seen in an outpatient clinic setting. This finding may be due to differences in the clinical presentation of EPL in these 2 settings and warrants further investigation.

### Limitations

Our study is limited by the accuracy of EPL diagnosis and procedure codes. Claims data are intended for billing purposes, and billing codes may be incomplete or inaccurate for our exposures and covariates of interest. Due to the nature of claims data, we did not know which facilities had mifepristone available and could not confirm whether misoprostol was used for EPL management vs another indication, such as management of retained products of conception. We could not accurately determine specific gestational durations, exact dosing or route of misoprostol administration, and time from mifepristone administration to misoprostol usage, all of which may influence effectiveness and outcomes with medication management. Additionally, this study was based on a large convenience sample of privately insured individuals. To ensure the generalizability of our findings, it may be helpful to repeat these analyses in a dataset that includes patients with public insurance or no insurance.

## Conclusions

The findings of this cohort study suggest that mifepristone remains underutilized for EPL management, but its use is associated with a lower need for subsequent uterine aspiration and a decrease in the number of subsequent visits to an ED. Continued efforts are needed to reduce barriers to mifepristone use for medication management of EPL. Increasing access to mifepristone for EPL management may decrease health care utilization and expenditures.

## Notes

Supplement 1. **eTable.** Diagnosis and Procedure Codes

Supplement 2.
Data Sharing Statement
