# Supplementary material for: Outcomes After Early Pregnancy Loss Management With Mifepristone Plus Misoprostol vs Misoprostol Alone
Source: JAMA Netw Open. 2024 Oct 8;7(10):e2435906. doi: 10.1001/jamanetworkopen.2024.35906 (PMC11581616; doi:10.1001/jamanetworkopen.2024.35906)
Supplement: Supplement 1. — eTable. Diagnosis and Procedure Codes [file jamanetwopen-e2435906-s001.pdf]

Supplementary Online Content

Benson LS, Gunaje N, Holt SK, Gore JL, Dalton VK. Outcomes of early pregnancy loss management with mifepristone and misoprostol vs misoprostol alone. *JAMA Netw Open*. 2024;7(10):e2435906. doi:10.1001/jamanetworkopen.2024.35906

eTable. Diagnosis and Procedure Codes

This supplementary material has been provided by the authors to give readers additional information about their work.

© 2024 Benson LS et al. *JAMA Network Open.*

eTable. Diagnosis and Procedure Codes

	ICD-10 diagnosis codes	CPT codes	ICD-10 procedure codes
Inclusion Criteria			
Early pregnancy loss	O02.1, O03.X		
Exclusion criteria			
Ectopic or molar pregnancy	O00.X, O01.X, O08.X		
Induced abortion	O04.X, Z33.2	59840, 59841, 59851	
Stillbirth	P95.X, Z37.1, Z37.4, Z37.7, O36.4, O36.4XX0, O36.4XX1, O36.4XX3, O36.4XX4, O36.4XX5, O36.4XX9		
Recent medication management of EPL	*Based on NDC codes for mifepristone and misoprostol*		
Recent surgical management of EPL		59820, 59812, 59821	0UDBxxx
Primary outcome			
Surgical management		59820, 59812, 59821	0UDBxxx
Medication management	*Based on NDC codes for mifepristone and misoprostol*		
Complications			
Inpatient hospitalization, EPL-related	O02.1, O03.X, N93.8, N93.9, O20.x		
Hemorrhage requiring blood transfusion		36430	30233xx
Uterine artery embolization		37241, 37243, 37244	04LE3xx
Other surgical management (laparoscopy or laparotomy)		49320, 49000, 58578	0WJGxxx, 0UQ9xxx
Cervical injury or laceration repair	O71.3	57720	0UQCxx
Uterine perforation or other GU injury	N99.7, N99.71		
Infection related to EPL	O03.0, O03.37, O03.5, O03.87		
Additional covariates			
Prior threatened abortion or bleeding in early pregnancy	O20.X, O26.85X		
Prenatal care in current pregnancy	Z34.X, O09.X		
History of infertility	N97.X, N46.X, O09.81X		

© 2024 Benson LS et al. *JAMA Network Open.*
