# Supplementary material for: Outcomes After Early Pregnancy Loss Management With Mifepristone Plus Misoprostol vs Misoprostol Alone
Source: JAMA Netw Open. 2024 Oct 8;7(10):e2435906. doi: 10.1001/jamanetworkopen.2024.35906 (PMC11581616; doi:10.1001/jamanetworkopen.2024.35906)
Supplement: Supplement 2. — Data Sharing Statement [file jamanetwopen-e2435906-s002.pdf]

Data Sharing Statement

Benson. Outcomes After Early Pregnancy Loss Management With Mifepristone Plus Misoprostol vs Misoprostol Alone. *JAMA Netw Open*. Published October 08, 2024. doi:10.1001/jamanetworkopen.2024.35906

Data
Data available: No

Additional Information
Explanation for why data not available: Our institutional contract with IBM MarketScan does not permit us to share our dataset publicly.
